# Incidence and Predictors of Acute Respiratory Distress Syndrome in Patients With Sepsis: A Two-Year Prospective Cohort Study From Khyber Pakhtunkhwa, Pakistan

**DOI:** 10.7759/cureus.108400

**Published:** 2026-05-06

**Authors:** Aamir Khan, Talal Hamid, Sachin Singh, Sana Afridi, Ikramullah Atal, Ameer Hamza, Anum Kashif, Momna Arif, Usama Asad Ullah, M Hassaan Shah, Naqeeb Ullah

**Affiliations:** 1 General Medicine, Khyber Teaching Hospital, Peshawar, PAK; 2 General Medicine, Abbottabad Medical Complex, Abbottabad, PAK; 3 Accident and Emergency Medicine, Bedford Hospital, Bedford, GBR; 4 General Medicine, District Headquarters Hospital, Haripur, PAK; 5 Internal Medicine, Khyber Teaching Hospital, Peshawar, PAK; 6 Pulmonology, Medical Teaching Institution Mardan Medical Complex, Mardan, PAK; 7 Internal Medicine, Central Park Teaching Hospital, Lahore, PAK; 8 Acute Internal Medicine, Midland Metropolitan University Hospital, Smethwick, GBR; 9 Internal Medicine, Lady Reading Hospital, Peshawar, PAK

**Keywords:** acute respiratory distress syndrome, incidence, pao₂/fio₂ ratio, predictors, sepsis, serum lactate, sofa score

## Abstract

Background: Sepsis is a potentially fatal illness that is often made worse by Acute Respiratory Distress Syndrome (ARDS), which increases morbidity and mortality.

Objective: To determine the incidence of acute respiratory distress syndrome (ARDS) and to identify its clinical and laboratory predictors (including serum lactate levels, partial pressure of oxygen/fraction of inspired oxygen {PaO_2_/FiO_2_} ratio, and the Sequential Organ Failure Assessment {SOFA} score) among adult patients with sepsis in a prospective observational cohort study.

Methodology: A prospective observational cohort study was conducted from March 2023 to February 2025. Following a sequential enrollment process, adult patients (≥18 years) with a diagnosis of sepsis based on Sepsis-3 criteria were observed daily for the onset of ARDS using the Berlin definition. Vital signs, test results, comorbidities, baseline demographics, and the source of infection were all documented. IBM SPSS Statistics for Windows, version 26 (IBM Corp., Armonk, NY, USA) was used to analyze the data. To find independent predictors of ARDS, univariate and multivariable logistic regression analyses were conducted.

Results: A total of 364 septic patients were included, of whom 108 (29.67%) developed ARDS. ARDS patients were older (mean age 57.48 ± 15.79 years), with 65 (60.19%) male patients. Comorbidities included diabetes mellitus (42, 38.89%), hypertension (35, 32.41%), and chronic kidney disease (18, 16.67%). Pulmonary infection was present in 62 (57.41%) of ARDS cases. ARDS patients had higher heart rate (107.23 vs. 100.45 bpm), respiratory rate (25.12 vs. 21.45/min), lower GCS (13.45 vs. 14.45), higher SOFA score (6.78 vs. 4.32), elevated WBC (16.34 vs. 12.34 ×10³/µL), and serum lactate (3.42 vs. 2.61 mmol/L). Independent predictors of ARDS were PaO_2_/FiO_2_ <200 mmHg, serum lactate >2 mmol/L, GCS <14, pulmonary infection, SOFA ≥6, and age ≥60 years.

Conclusion: Approximately one-third of septic patients developed ARDS, with severity of illness and pulmonary involvement being key predictors.

## Introduction

Sepsis refers to a life-threatening dysfunction of the various body organs due to an unregulated host response to an infection and has continued to be the most critical morbidity and mortality in the world [1,2]. In spite of the improvement of antimicrobial therapy, hemodynamic support, as well as critical care management, sepsis still poses a significant burden on healthcare systems, especially in low- and middle-income countries [3]. Some of the worst complications of it include acute respiratory failure, which often presents itself in the form of the Acute Respiratory Distress Syndrome (ARDS) [4]. ARDS is a disease that can be distinguished by diffuse alveolar injury, pulmonary vascular hyperpermeability, refractory hypoxemia, and bilateral pulmonary infiltrates that cannot be completely attributed to cardiac insufficiency or excessive fluid buildup [5].

Sepsis and ARD are closely associated, and sepsis is the most prevalent precipitant in this case [6]. Sepsis induces a systemic inflammatory cascade that causes endothelial injury, release of cytokines, neutrophil activation, and breakdown of the alveolar-capillary barrier, resulting in non-cardiogenic pulmonary edema [7]. The given pathophysiological interaction leads to prolonged mechanical ventilation, prolonged intensive care unit (ICU) stay, and high mortality rates [8]. ARDS is also linked to long-term pulmonary dysfunction, poor quality of life, and high cost of healthcare, even among the survivors [9].

Incidence of ARDS has been reported to be widely variable in the literature of studies due to varying patient populations, diagnostic conditions, time of assessment, and setting of health care [10]. The definition of ARDS in Berlin has standardized the diagnosis of the disease in relation to the onset time, chest X-rays, edema source, and level of hypoxemia (partial pressure of oxygen/fraction of inspired oxygen {PaO_2_/FiO_2_} ratio) [11]. But there is still variability in the clinical recognition. Moreover, not every patient with sepsis has ARDS, and it may be assumed that host-related factors, comorbid diseases, illness severity, laboratory parameters, and treatment-related variables can contribute to the susceptibility. The early predictors are important in risk stratification and early adoption of lung-protective measures [12].

Sepsis remains a major global health concern and a leading cause of morbidity and mortality, particularly in low- and middle-income countries such as Pakistan, where resource limitations further complicate outcomes. ARDS is a frequent and severe complication of sepsis, with international studies reporting an incidence of approximately 30-35% among septic patients and significantly higher associated mortality rates [13,14]. Despite this substantial burden, there is a paucity of local data from Pakistan regarding the incidence and predictors of ARDS in sepsis. Given the variability in patient characteristics, healthcare infrastructure, and disease patterns, region-specific evidence is essential for accurate risk stratification and early intervention. Therefore, this study was conducted to determine the incidence and identify the clinical and laboratory predictors of ARDS among patients diagnosed with sepsis in a tertiary care setting in Pakistan.

## Materials and methods

Study design and setting

This prospective observational cohort study was conducted at Bacha Khan Medical College (BKMC), Mardan Medical Complex, Mardan, a tertiary care teaching hospital in Khyber Pakhtunkhwa, Pakistan, equipped with a fully functional intensive care unit (ICU) that follows standard critical care protocols and evidence-based management guidelines. The study was carried out over a two-year period from March 2023 to February 2025. Adult patients (≥18 years) hospitalized with a diagnosis of sepsis were consecutively recruited and observed throughout their hospital stay for the development of ARDS. Sepsis was defined according to the Sepsis-3 criteria as suspected or confirmed infection accompanied by an acute increase of ≥2 points in the Sequential Organ Failure Assessment (SOFA) score, reflecting organ dysfunction [15,16]. ARDS was diagnosed based on the Berlin definition, requiring onset within one week of a known clinical insult, bilateral pulmonary opacities on chest imaging not fully explained by cardiac failure or fluid overload, and hypoxemia defined by PaO_2_/FiO_2_ ratio (mild: 200-300 mmHg, moderate: 100-200 mmHg, severe: <100 mmHg) with a minimum positive end-expiratory pressure (PEEP) of 5 cm H_2_O.

Inclusion and exclusion criteria

Adult patients (≥18 years) admitted with a diagnosis of sepsis, defined according to Sepsis-3 criteria, were eligible for inclusion. Consecutive sampling was employed, and patients admitted to medical wards or the intensive care unit (ICU) during the study period were prospectively enrolled. Patients referred from other hospital units (e.g., surgical ICU) or external healthcare facilities were also included, provided they fulfilled Sepsis-3 criteria at the time of admission to our center and had not developed ARDS prior to enrollment, ensuring uniform baseline assessment and follow-up.

Patients were excluded if they had pre-existing chronic pulmonary diseases associated with baseline hypoxemia (e.g., advanced COPD, interstitial lung disease), cardiogenic pulmonary edema (based on clinical and echocardiographic evaluation), or a prior history of ARDS before the current hospitalization. Additionally, patients with incomplete medical records or those who left against medical advice before adequate evaluation and follow-up were excluded from the analysis.

Sample size

The sample size was calculated using the single population proportion formula,

$n = \frac{Z^{2} \, p \, (1 - p)}{d^{2}}$.

In the above equation, n represents the required sample size, Z is the standard normal deviate corresponding to the desired confidence level (1.96 for a 95% confidence interval), p is the anticipated proportion (incidence) of ARDS among septic patients, and d is the margin of error (precision).

The end result was a sample of 364 patients after adding a 10% non-response allowance to the sample size. The non-probability consecutive (convenience) sampling method was used, in which all patients who fitted the inclusion criteria in the study period were recruited to the study until the required sample size was attained. Based on the anticipated number of ARDS events (~105-110), up to 10 predictor variables were included in the multivariable logistic regression model, following standard events-per-variable recommendations.

Data collection

A structured and predesigned proforma (see Appendices) was used for data collection, developed in consultation with senior consultants from the Departments of Medicine and Critical Care. Baseline information, including demographic characteristics, comorbidities, and source of infection, was recorded at admission. Clinical variables such as vital signs, Glasgow Coma Scale (GCS) score, and severity indices (including SOFA score) were documented systematically. Laboratory investigations included complete blood count, liver and renal function tests, serum lactate, inflammatory markers, and arterial blood gas (ABG) analysis.

Patients were monitored daily throughout hospitalization for the development of ARDS. The primary outcome was the incidence of ARDS among patients with sepsis, while secondary outcomes included identification of clinical and laboratory predictors, requirement for mechanical ventilation, duration of ICU stay, and in-hospital mortality. For patients who developed ARDS, the timing of onset, PaO_2_/FiO_2_ ratio, ventilatory parameters, and radiological findings were recorded in a standardized manner. ABG measurements were obtained at admission and during clinical deterioration, with values at the time of ARDS diagnosis used for analysis. Mechanical ventilation was administered according to standard ICU protocols, including lung-protective strategies where applicable. All laboratory analyses were performed in the hospital’s central laboratory following standard calibration procedures. Clinical assessments, including GCS and SOFA scoring, were conducted by trained physicians to ensure consistency. Cases with incomplete key variables were excluded, and complete-case analysis was performed.

Statistical analysis

Data were entered and analyzed using IBM SPSS Statistics for Windows, version 26 (IBM Corp., Armonk, NY, USA). The continuous variables were given in terms of mean + SD or median with interquartile range, as per the nature of the data, whereas categorical variables were given in terms of frequencies and percentages. The ARDS occurrence in the septic patients was calculated. The Chi-square test was used to perform univariate analysis. Variables that were found to have a p-value of 0.05 or less on univariate analysis were included in the multivariate logistic regression to determine independent predictors of ARDS. Adjusted odds ratios (AORs) with 95% confidence intervals (CIs) were reported, and multicollinearity among variables was assessed prior to model inclusion. The level of p-value (less than 0.05) was regarded as statistically significant.

Ethical approval

The study was started after receiving the ethical approval of the Ethical Review Board, BKMC, Mardan Medical Complex, Mardan (Approval No.: 924/BKMC, dated February 20, 2023). Patients or their representatives, who had the legal authority to sign informed consent, did so in writing. Patient information was kept confidential, and the study was done within the confines of the current ethical principles of conducting biomedical research involving human subjects.

## Results

Table 1 summarizes 364 septic patients, including 108 who developed ARDS and 256 who did not. Patients with ARDS were older (mean 57.48 ± 15.79 years vs. 52.87 ± 16.32) and included 60.19% males. Comorbidities were more frequent in the ARDS group, with diabetes (38.89% vs. 32.03%), hypertension (32.41% vs. 24.61%), and chronic kidney disease (16.67% vs. 10.94%). Pulmonary infections predominated among ARDS patients (57.41% vs. 31.25%). Vital signs reflected higher severity in ARDS, with elevated heart rate (107.23 vs. 100.45 bpm), respiratory rate (25.12 vs. 21.45/min), lower GCS (13.45 vs. 14.45), and higher SOFA score (6.78 vs. 4.32). Laboratory values indicated greater systemic involvement, including higher WBC (16.34 vs. 12.34 ×10³/µL), lactate (3.42 vs. 2.61 mmol/L), CRP (94.32 vs. 70.41 mg/L), and lower PaO_2_/FiO_2_ ratio (148.23 vs. 282.45 mmHg). Median hospital stay was longer for ARDS patients (14 vs. 9 days).

**Table 1:** Baseline demographic, clinical, and laboratory characteristics of study population (n = 364) ARDS, acute respiratory distress syndrome; SBP, systolic blood pressure; DBP, diastolic blood pressure; GCS, Glasgow Coma Scale; SOFA, Sequential Organ Failure Assessment; CRP, C-reactive protein; PaO_2_, partial pressure of oxygen; FiO_2_, fraction of inspired oxygen; ALT, alanine aminotransferase; AST, aspartate aminotransferase; IQR, interquartile range

Category	Variable	ARDS (n = 108)	No ARDS (n = 256)
Demographics	Age (years), mean ± SD	57.48 ± 15.79	52.87 ± 16.32
	Male, n (%)	65 (60.19)	147 (57.42)
	Female, n (%)	43 (39.81)	109 (42.58)
Comorbidities	Diabetes mellitus, n (%)	42 (38.89)	82 (32.03)
	Hypertension, n (%)	35 (32.41)	63 (24.61)
	Chronic kidney disease, n (%)	18 (16.67)	28 (10.94)
Source of Infection	Pulmonary, n (%)	62 (57.41)	80 (31.25)
	Urinary, n (%)	22 (20.37)	70 (27.34)
	Abdominal, n (%)	14 (12.96)	58 (22.66)
	Other, n (%)	10 (9.26)	48 (18.75)
Vital signs at admission	Mean SBP (mmHg)	108.34 ± 17.21	114.23 ± 18.45
	Mean DBP (mmHg)	70.12 ± 11.87	73.51 ± 12.59
	Heart rate (bpm)	107.23 ± 19.12	100.45 ± 17.89
	Respiratory rate (per min)	25.12 ± 5.67	21.45 ± 4.78
	Temperature (°C)	38.48 ± 1.18	38.15 ± 1.05
	Glasgow Coma Scale (GCS), mean ± SD	13.45 ± 1.78	14.45 ± 0.89
	SOFA score, mean ± SD	6.78 ± 2.56	4.32 ± 1.87
Laboratory parameters at admission	WBC (×10³/µL)	16.34 ± 6.21	12.34 ± 4.12
	Serum lactate (mmol/L)	3.42 ± 1.48	2.61 ± 1.03
	Creatinine (mg/dL)	1.78 ± 0.72	1.28 ± 0.54
	CRP (mg/L)	94.32 ± 41.56	70.41 ± 32.18
	PaO_2_/FiO_2_ ratio (mmHg)	148.23 ± 39.12	282.45 ± 52.67
	ALT (U/L)	46.78 ± 20.12	40.12 ± 17.45
	AST (U/L)	42.23 ± 18.34	36.45 ± 15.67
	Bilirubin (mg/dL)	1.32 ± 0.68	1.05 ± 0.49
Median hospital stay	(days, IQR)	14 (10–18)	9 (6–12)

**Figure 1:**
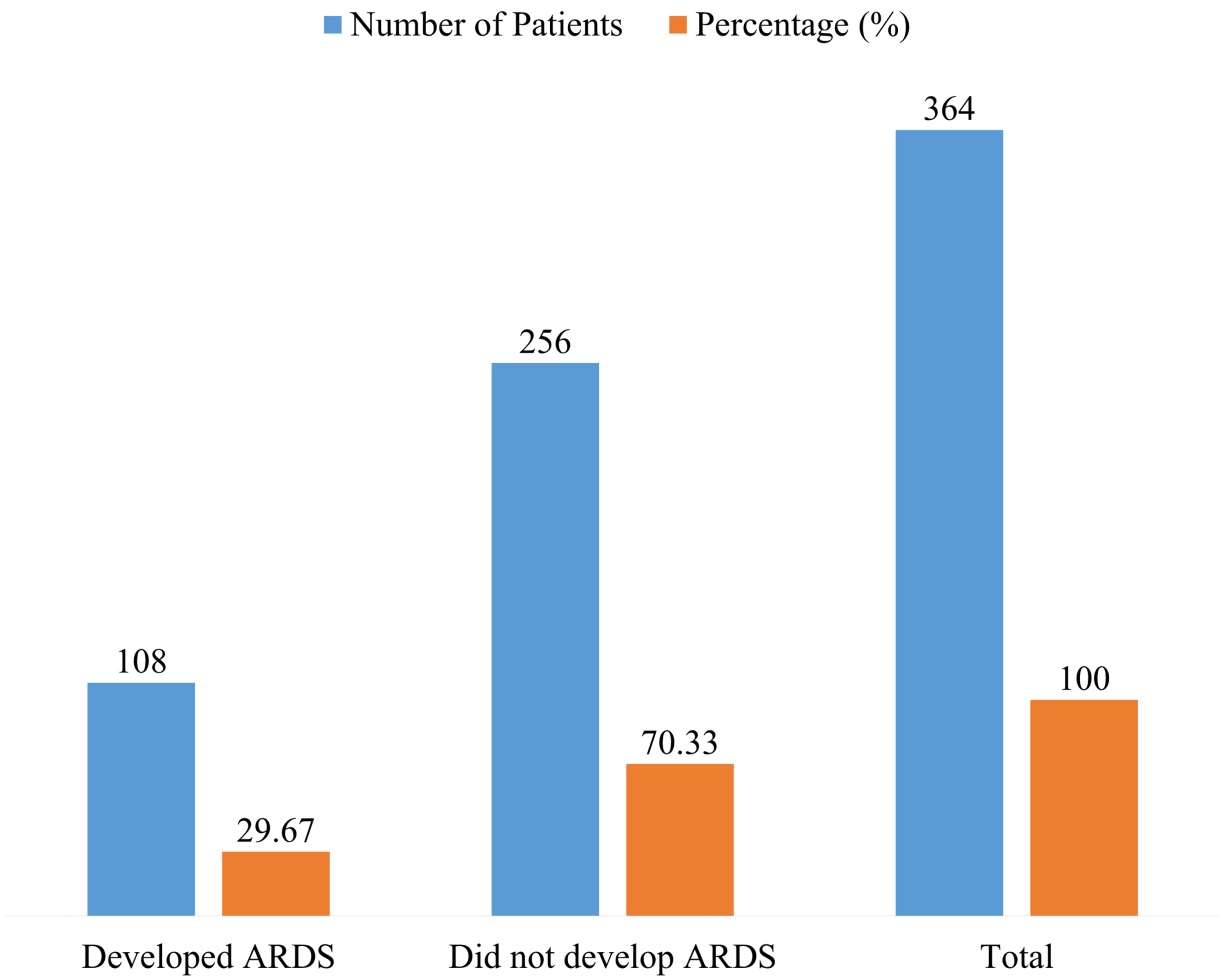
Incidence of ARDS ARDS, acute respiratory distress syndrome

Among the 364 septic patients, 108 (29.67%) developed ARDS while 256 (70.33%) did not, highlighting that nearly one-third of septic patients in this cohort experienced this severe complication (Figure 1).

**Figure 2:**
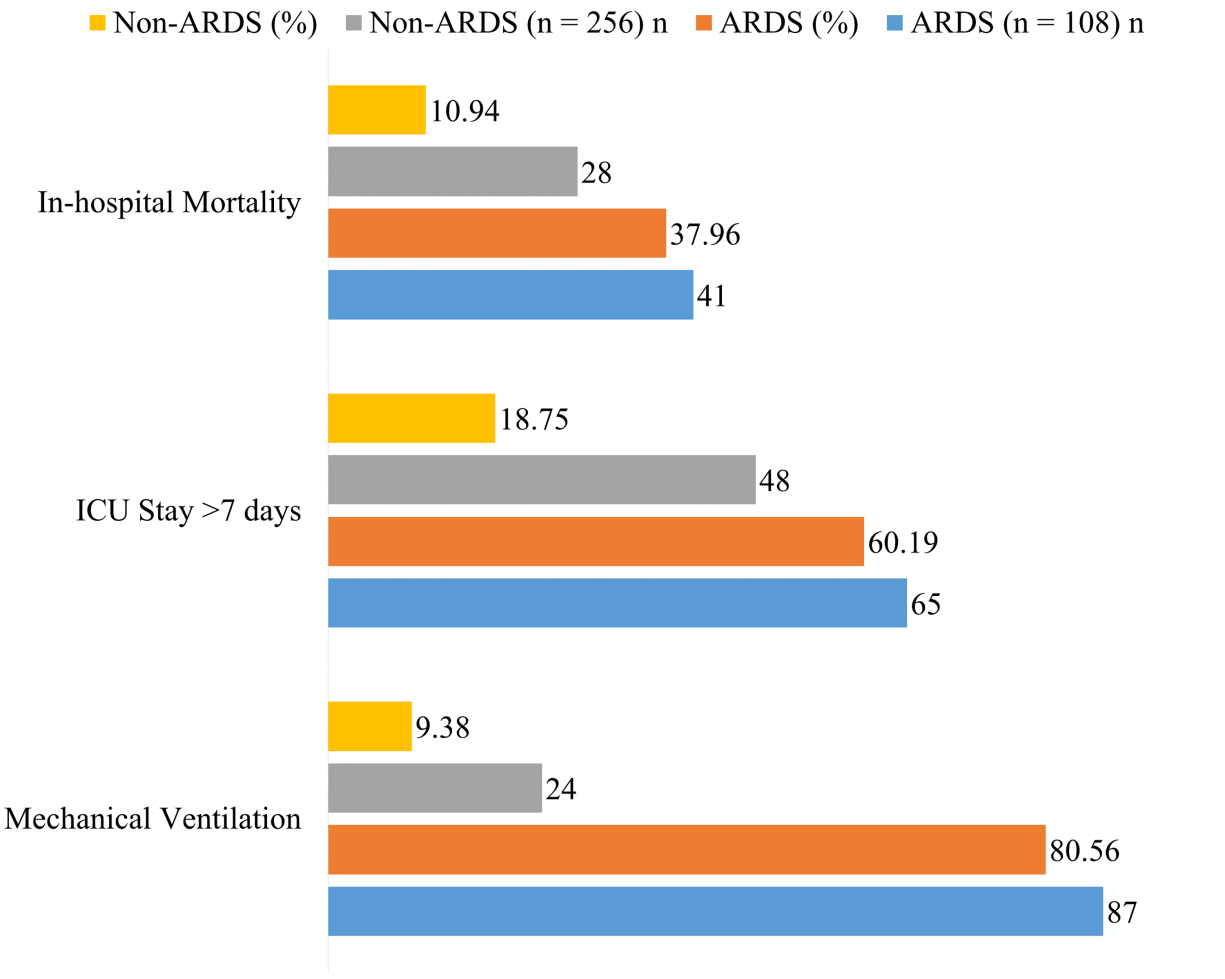
Clinical outcomes in ARDS vs. non-ARDS patients (n = 364) ARDS, acute respiratory distress syndrome

ARDS patients had markedly worse outcomes; 80.56% required mechanical ventilation compared to 9.38% in non-ARDS patients, 60.19% had ICU stay >7 days versus 18.75%, and in-hospital mortality was higher at 37.96% compared to 10.94% in non-ARDS patients (Figure 2).

Table 2 summarizes the univariate analysis of potential predictors of ARDS among 364 septic patients. Male gender was observed in 65 (60.19%) of ARDS patients and 147 (57.42%) of non-ARDS patients, showing no significant association (p = 0.63). Age ≥60 years was more frequent in the ARDS group (52, 48.15%) than in the non-ARDS group (78, 30.47%; p = 0.003). Comorbidities such as diabetes mellitus (42, 38.89% vs. 82, 32.03%; p = 0.20) and hypertension (35, 32.41% vs. 63, 24.61%; p = 0.11) were not statistically significant. Pulmonary infection was strongly associated with ARDS (62, 57.41% vs. 80, 31.25%; p < 0.001). Laboratory and clinical markers including serum lactate >2 mmol/L (79, 73.15% vs. 109, 42.58%; p < 0.001), PaO_2_/FiO_2_ <200 mmHg (65, 60.19% vs. 28, 10.94%; p < 0.001), SOFA score ≥6 (58, 53.70% vs. 38, 14.84%; p < 0.001), and GCS <14 (46, 42.59% vs. 18, 7.03%; p < 0.001) were significantly higher in the ARDS group, indicating their potential role as predictors of ARDS development.

**Table 2:** Univariate analysis of predictors of ARDS ARDS, acute respiratory distress syndrome; GCS, Glasgow Coma Scale; SOFA, Sequential Organ Failure Assessment; PaO_2_, partial pressure of oxygen; FiO_2_, fraction of inspired oxygen

Variable	ARDS n (%)	No ARDS n (%)	p-value
Male	65 (60.19)	147 (57.42)	0.63
Female	43 (39.81)	109 (42.58)	
Age ≥60 years	52 (48.15)	78 (30.47)	0.003
Diabetes mellitus	42 (38.89)	82 (32.03)	0.20
Hypertension	35 (32.41)	63 (24.61)	0.11
Pulmonary infection	62 (57.41)	80 (31.25)	<0.001
Serum lactate >2 mmol/L	79 (73.15)	109 (42.58)	<0.001
PaO_2_/FiO_2_ <200 mmHg	65 (60.19)	28 (10.94)	<0.001
SOFA ≥6	58 (53.70)	38 (14.84)	<0.001
GCS <14	46 (42.59)	18 (7.03)	<0.001

Independent predictors of ARDS included PaO_2_/FiO_2_ <200 mmHg (AOR 7.14), serum lactate >2 mmol/L (AOR 3.25), GCS <14 (AOR 3.12), pulmonary infection (AOR 2.87), SOFA ≥6 (AOR 2.95), and age ≥60 years (AOR 1.98). Chronic kidney disease was not statistically significant (AOR 1.72, p=0.11) (Table 3).

**Table 3:** Multivariable logistic regression for independent predictors of ARDS ARDS, acute respiratory distress syndrome; GCS, Glasgow Coma Scale; SOFA, Sequential Organ Failure Assessment; PaO_2_, partial pressure of oxygen; FiO_2_, fraction of inspired oxygen

Predictor	Adjusted odds ratio (AOR)	95% CI	p-value
Age ≥60 years	1.98	1.15–3.42	0.014
Pulmonary infection	2.87	1.65–4.99	<0.001
Serum lactate >2 mmol/L	3.25	1.89–5.57	<0.001
PaO_2_/FiO_2_ <200 mmHg	7.14	3.76–13.55	<0.001
SOFA ≥6	2.95	1.72–5.08	<0.001
GCS <14	3.12	1.68–5.80	<0.001
Chronic kidney disease	1.72	0.88–3.34	0.11

## Discussion

This prospective cohort study included 364 septic patients, among whom 108 (29.67%) developed ARDS during hospitalization. This incidence is consistent with global evidence, as a recent meta-analysis of 24 studies reported an overall ARDS incidence of approximately 34% among patients with sepsis, indicating that nearly one-third of septic patients may develop this life-threatening complication across diverse clinical settings [13]. Although our observed incidence is slightly lower than pooled estimates, it reinforces the well-established understanding that ARDS remains a frequent and severe complication of sepsis.

In the context of Pakistan, there is limited but emerging evidence addressing ARDS in critically ill septic patients. Previous regional ICU-based studies from Pakistan have reported variable ARDS frequencies ranging from approximately 25% to 40% in critically ill and septic populations, highlighting substantial disease burden and variability across healthcare settings due to differences in diagnostic practices, ICU resources, and patient profiles [17,18]. However, these studies remain scarce and are often limited to single-center ICU populations, underscoring the need for more standardized prospective data such as the present study. Our findings, therefore, contribute important local evidence from a tertiary care hospital in Khyber Pakhtunkhwa, which may help inform future regional risk stratification strategies and support the development of evidence-based critical care protocols to reduce ARDS-related morbidity and mortality in septic patients.

We observed significant differences in clinical outcomes between ARDS and non-ARDS patients. Mechanical ventilation was required in 80.56% of ARDS patients compared to 9.38% of non-ARDS patients, while prolonged ICU stay (>7 days) was observed in 60.19% versus 18.75%, respectively. In-hospital mortality was also markedly higher in the ARDS group (37.96% vs. 10.94%). These findings are consistent with previous studies demonstrating that sepsis-associated ARDS is strongly linked with increased mortality, prolonged ventilation, and greater ICU resource utilization, particularly in patients with severe hypoxemia and multiorgan dysfunction [14]. Collectively, these results emphasize the need for early identification of high-risk septic patients to enable the timely implementation of lung-protective strategies and intensive monitoring.

Our univariate results showed that age ≥60 years (48.15% vs. 30.47, p=0.003), pulmonary infections (57.41% vs. 31.25, p<0.001), high serum lactate (>2 mmol/L) (73.15% vs. 42.58, p<0.001), PaO_2_/FiO_2_ <200 mmHg (60.19% Such outcomes underscore the severity of illness indicators and respiratory impairment as a major distinction between the ones coming to ARDS and the ones who do not. The role of the acute severity indices compared with the baseline comorbidities has also been proposed as a predictor of ARDS in sepsis [19], with similar findings indicating that the SOFA score and pulmonary infection are reliable predictors of ARDS in sepsis elsewhere.

PaO_2_/FiO_2_ less than 200 mmHg, serum lactate more than 2 mmol/L, GCS less than 14, pulmonary infection, SOFA greater than 6, and age greater than 60 years turned out to be the strongest independent predictors in multivariate logistic regression (PaO_2_/FiO_2_, 3.25; serum lactate, 3.12; GCS, 3.04; pulmonary infection, 2.87). Such results are reflected in the existing literature that highlights reduced oxygenation and organ dysfunction indicators as critically important in sepsis ARDS pathogenesis, in line with meta-analytic demonstrations of higher SOFA and severity scores reflecting higher ARDS risk [13].

A smaller number of older studies have demonstrated differing associations with demographic characteristics and comorbidity factors. Indicatively, Fein et al. (1983) were able to observe that shock and thrombocytopenia were higher among patients with ARDS and who were in septicemia, and that there was no significant difference in terms of age and gender, as the studies indicated the heterogeneity of risk profiles based on patient groups and diagnostic conditions [20]. We find that our results are consistent with the larger trend, which shows that acute physiologic derangements are more predictive of ARDS than chronic baseline considerations are.

Lastly, the presence of serum biomarkers (lactate and inflammatory markers) in our study was consistent with other observational data indicating that elevated CRP levels and metabolic derangements were associated with an increased risk of ARDS in patients with sepsis [21]. Although the specific biomarker panels varied across studies, the overall trend of an exaggerated inflammatory response contributing to ARDS development supported the need to integrate clinical and biochemical parameters for early risk stratification.

Study strengths and limitations

This study has several important strengths. Its prospective cohort design allowed systematic and real-time monitoring of 364 septic patients, enabling accurate identification of ARDS onset through daily clinical assessment. The use of standardized diagnostic criteria, including Sepsis-3 for sepsis and the Berlin definition for ARDS, enhances the validity and comparability of findings with international literature. Additionally, the comprehensive collection of demographic, clinical, laboratory, and outcome variables facilitated robust multivariable logistic regression analysis to identify independent predictors of ARDS. The inclusion of clearly defined primary and secondary outcomes further strengthens the methodological rigor and clinical relevance of the study.

However, certain limitations should be acknowledged. Being a single-center study conducted at a tertiary care hospital, the findings may have limited generalizability to other healthcare settings with different patient populations and resource availability. The use of non-probability consecutive (convenience) sampling may introduce selection bias. Furthermore, although key clinical and laboratory parameters were analyzed, the study did not include advanced inflammatory biomarkers or genetic factors that may influence ARDS susceptibility. Long-term outcomes beyond hospital discharge were also not assessed. These limitations highlight areas for future multicenter studies and more comprehensive investigations.

## Conclusions

Almost one-third of the patients in this group of septic patients acquired ARDS, and with a significantly poorer outcome, such as increased mechanical ventilation, extended ICU admission, and in-hospital mortality. There were independent predictors of ARDS, which were: PaO_2_/FiO_2_ less than 200 mmHg, serum lactate above 2 mmol/L, GCS less than 14, pulmonary infection, SOFA at least 6, and age at least 60 years. The findings indicate that early recognition and risk stratification of sepsis, along with acute physiological derangements and infection severity, are stronger predictors of ARDS development than underlying comorbidities.

## Appendices

**Table 4:** Structured data collection proforma ABG, arterial blood gas; ALT, alanine aminotransferase; ARDS, acute respiratory distress syndrome; AST, aspartate aminotransferase; BP, blood pressure; CRP, C-reactive protein; FiO_2_, fraction of inspired oxygen; GCS, Glasgow Coma Scale; ICU, intensive care unit; PaO_2_, partial pressure of oxygen; PEEP, positive end-expiratory pressure; SOFA, Sequential Organ Failure Assessment; WBC, white blood cell count

Section	Variable	Details/Options
A. General Information	Serial Number	__________
	Date of Admission	__________
	Patient ID	__________
B. Demographics	Age (years)	__________
	Gender	☐ Male ☐ Female
C. Admission Details	Admission Location	☐ Medical Ward ☐ ICU ☐ Referred from Surgical ICU ☐ Referred from Other Center
	Source of Referral	☐ Direct Admission ☐ Internal Referral ☐ External Referral
D. Sepsis Assessment (Sepsis-3)	Suspected/Confirmed Infection	☐ Yes ☐ No
	SOFA Score at Admission	__________
	SOFA ≥2	☐ Yes ☐ No
E. Comorbidities	Diabetes Mellitus	☐ Yes ☐ No
	Hypertension	☐ Yes ☐ No
	Chronic Kidney Disease	☐ Yes ☐ No
	Chronic Pulmonary Disease	☐ Yes ☐ No (If yes → specify: __________)
	Other Comorbidities	☐ Yes ☐ No (Specify: __________)
F. Source of Infection	Primary Source	☐ Pulmonary ☐ Urinary ☐ Abdominal ☐ Bloodstream ☐ Other (specify: __________)
G. Vital Signs at Admission	Systolic BP (mmHg)	__________
	Diastolic BP (mmHg)	__________
	Heart Rate (bpm)	__________
	Respiratory Rate (/min)	__________
	Temperature (°C)	__________
H. Neurological Status	Glasgow Coma Scale (GCS)	__________
	GCS <14	☐ Yes ☐ No
I. Laboratory Parameters	WBC (×10³/µL)	__________
	Serum Lactate (mmol/L)	__________
	Lactate >2 mmol/L	☐ Yes ☐ No
	Creatinine (mg/dL)	__________
	CRP (mg/L)	__________
	ALT (U/L)	__________
	AST (U/L)	__________
	Bilirubin (mg/dL)	__________
J. Arterial Blood Gas (ABG)	PaO_2_ (mmHg)	__________
	FiO_2_ (%)	__________
	PaO_2_/FiO_2_ Ratio	__________
	PaO_2_/FiO_2_ <200	☐ Yes ☐ No
K. ARDS Monitoring (Berlin Criteria)	ARDS Developed	☐ Yes ☐ No
	Time of Onset	☐ ≤1 week ☐ >1 week
	Chest Imaging	☐ Bilateral Opacities ☐ Not Present
	Edema Origin	☐ Non-cardiogenic ☐ Cardiogenic Excluded
	ARDS Severity	☐ Mild (200–300) ☐ Moderate (100–200) ☐ Severe (<100)
	PEEP ≥5 cm H_2_O	☐ Yes ☐ No
L. Ventilatory Support	Mechanical Ventilation Required	☐ Yes ☐ No
	Type	☐ Invasive ☐ Non-invasive
M. ICU Course	ICU Admission	☐ Yes ☐ No
	ICU Stay Duration (days)	__________
	ICU Stay >7 days	☐ Yes ☐ No
N. Outcomes	Hospital Stay (days)	__________
	In-Hospital Mortality	☐ Yes ☐ No
	Discharge Status	☐ Recovered ☐ Referred ☐ Deceased
